# Additive value of repeat transthoracic echocardiography for excluding infective endocarditis in patients with *Staphylococcus aureus* bacteremia

**DOI:** 10.1186/s44348-025-00061-6

**Published:** 2026-02-10

**Authors:** Rami M. Abazid, Osama Smettei, Sameh Awadallah, Adel Widyan, Nicole Wuzynski, Mohamed Hashem Nabhan, Mohamed M. Ibrahim, Magdi Hassanin, Andrew Mathew, Sabe De, Rodrigo Bagur, Nikolaos Tzemos

**Affiliations:** 1 Division of Cardiology, Department of Medicine, Sault Area Hospital, Northern Ontario School of Medicine University, Sault Ste Marie, ON Canada; 2 Division of Cardiology, Department of Medicine, London Health Sciences Centre, University Hospital, London, ON Canada; 3 Department of Mathematics, College of Science, Qassim University, Qassim, Saudi Arabia; 4 Poznan University of Medical Science, Poznan, Poland; 5 Faculty of Health Science, Western University, London, ON Canada

**Keywords:** Transesophageal echocardiography, Infective endocarditis, *Staphylococcus aureus*, Bacteremia, Transthoracic echocardiogram

## Abstract

**Background:**

We aim to analyze the additive value of repeated transthoracic echocardiography (TTE) within a 1-week interval after a baseline TTE to diagnose infective endocarditis (IE) in patients admitted with *Staphylococcus aureus* bacteremia (SAB).

**Methods:**

We prospectively enrolled consecutive patients with SAB who were referred for TTE and transesophageal echocardiography (TEE) to exclude IE between January 2017 to December 2019. All patients underwent a second TTE within 5 to 7 days. We excluded patients with poor echo windows, previous IE, valve repair/replacement, and those with cardiac devices or a dialysis catheter in place.

**Results:**

A total of 105 patients were enrolled, of which 40 (38.1%) were female. The mean age was 52 ± 14 years. Sixty-four patients (61%) had a defined source of infection, and 36 (34.3%) were intravenous drug users. The majority (n = 74, 70.5%), had methicillin-sensitive *S. aureus*. Sixteen patients (15.2%) were diagnosed with definite IE based on TEE findings as follows: eight tricuspid valve IE, four mitral valve IE, three aortic valve IE, and one with double valve IE (mitral and tricuspid). The mortality rate was 7.6% (two patients with definite IE and six without IE). Vegetations were not detected in one patient on the first TTE, compared to TEE and the second TTE. The baseline TTE had a sensitivity of 93.8%, specificity of 87.6% and accuracy of 88.6% in identifying echocardiographic evidence of IE. The addition of second TTE findings increased the sensitivity to 100%, specificity to 95.5%, and diagnostic accuracy to 96.2% in comparison to TEE for the detection of IE.

**Conclusions:**

A repeat TTE within 5 to 7 days of an initial study significantly enhances diagnostic accuracy for detecting IE in patients with SAB and may help reduce the need for TEE in selected low-risk cases.

## Background

*Staphylococcus aureus* is the most common cause of infective endocarditis (IE) and is associated with a high morbidity and mortality [1, 2]. Prompt identification of valvular and other cardiac involvement is crucial for appropriate treatment to reduce the incidence of valvular destruction, peripheral embolization, and to improve outcomes [1]. Echocardiography is the imaging modality of choice to diagnose IE through the detection of vegetations, valvular damage, and other cardiac lesions. Although transthoracic echocardiography (TTE) is the initial modality used to diagnose IE, it has diagnostic limitations. Transoesophageal echocardiography (TEE), with its high spatial resolution, can detect small pathologies more effectively. Multiple reports have demonstrated that TEE has a significantly higher sensitivity (95%) than TTE (61%) in diagnosing IE [1, 3, 4].

Importantly, the diagnostic performance of echocardiograms is highly influenced by clinical findings and the pretest probability of IE. Therefore, identifying high-risk features of IE, such as the presence of peripheral embolization or a cardiac murmur, can guide management decisions in patients with suspected IE [5–7]. Additionally, several scoring systems have been developed to assist in the management of patients with suspected IE related to *S. aureus* bacteremia (SAB) [8, 9]. For instance, the VIRSTA score is used to assess IE risk within 48 h of a SAB diagnosis. Patients with a VIRSTA score of ≥ 3 are strongly recommended to undergo an urgent echocardiographic study [9]. In patients with SAB and suspicion of IE, the conventional approach is to perform a baseline TTE as soon as possible to confirm IE diagnosis, assess complications, and guide the management plan. If the initial scan is normal or inconclusive, the IE international working group recommends performing a repeat TTE within a short time interval of 5 to 7 days [1, 10]. A previous retrospective study indicated that conducting second and third TTEs provided additional diagnostic information in approximately 27% of patients with suspected IE [11]. International guidelines for the diagnosis and management of IE acknowledge the importance of repeat imaging and recommend early use of TEE when clinical suspicion remains high. However, they lack strong evidence to support a specific timeline or clearly establish the diagnostic performance of repeated TTE [1, 2, 10]. To date, no prospective studies have validated the recommendation to repeat TTE, leaving the optimal timing and frequency of echocardiographic assessments uncertain and a subject of ongoing investigation. In this study, we aim to prospectively explore the additive diagnostic value of performing a repeat TTE within a 1-week interval (range, 5–7 days) in the identification and exclusion of IE among patients admitted with SAB.

## Methods

### Ethics statement

The study protocol was approved by the Lawson Research Institute, and all participants provided informed consent to take part in the study.

### Study design and population

**Fig. 1:**
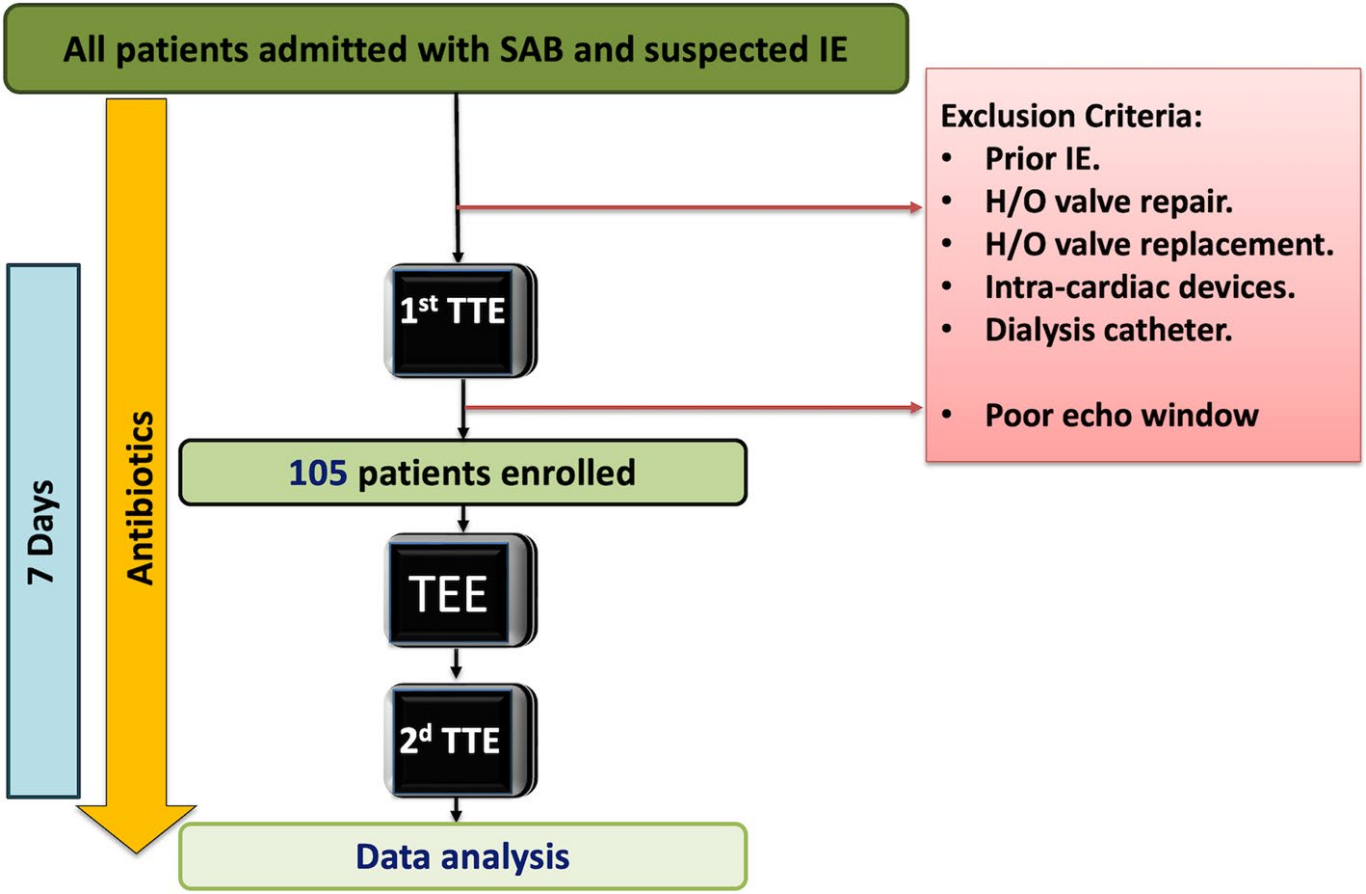
Flowchart demonstrating the inclusion of patients in the study. H/O, history of; IE, infective endocarditis; SAB, *Staphylococcus aureus* bacteremia; TEE, transoesophageal echocardiography; TTE, transthoracic echocardiography

We prospectively enrolled consecutive patients who were admitted to hospital with SAB and persistent fever defined as a temperature ≥ 38.0 °C lasting more than 5 days despite antibiotic therapy [12]. Patients with suspected IE, as defined by the modified Duke criteria, were referred for echocardiography imaging to rule out the diagnosis. Clinical suspicion of IE was based on one or more of the following: persistent bacteremia, embolic events, a new heart murmur, or other suggestive features such as vascular or immunologic phenomena [8]. We excluded patients with previous IE, valve repair or replacement, patients with cardiac devices, or with a dialysis catheter, as shown in Fig. 1.

### Echocardiography

All patients underwent initial TTE and TEE using an EPIQ ultrasound machine (Philips Medical Systems). Standard TTE views recommended by the American Society of Echocardiography [13] were obtained, along with off-axis views if necessary to assess for evidence of IE such as vegetation (oscillating, irregularly-shaped echo densities attached to a valvular, subvalvular apparatus, intracardiac structures, or mural endocardium), destructive valvular lesion, valvular perforation, perivalvular abscess, or fistula [14]. Vegetation size was measured in the largest dimension. TEE was performed for all patients within 7 days of the initial TTE to assess heart valves and other cardiac structures as recommended by the American Society of Echocardiography guidelines [14]. Vegetation dimensions were reported in the same way as TTE measurements. TEE findings were used as the gold standard for comparison with TTEs results. A detailed second TTE was performed on all patients during their hospitalization with the same echo machines within 5 to 7 days of the initial TTE scan. The follow-up TTE and TEE studies were interpreted in a blinded fashion by two experienced cardiologists (RMA and OS). Any discrepancies between the two readings were adjudicated by a third reviewer (NT), who issued the final decision.

### Clinical follow-up

Patients were followed during hospital admission and via a phone call 3 months after hospital discharge. IE complications, surgical intervention, or deaths were reported. All patients continued receiving antibiotics while waiting for the second TTE. The decision regarding antibiotic coverage was based on a shared decision between an infectious disease specialist, the primary physician, and the cardiologist in case of confirmed IE. The duration of antibiotic courses depended on the final diagnosis, the source of infection, and the progression of the clinical scenario.

### Statistical analysis

Continuous variables were represented as means/standard deviations, and categorical variables were represented as numbers and percentages. We used the chi-square test to measure sensitivity, specificity, positive predictive value, negative predictive value (NPV), positive likelihood ratio, negative likelihood ratio, and accuracy of the first and second TTEs in identifying any evidence of IE. Statistical analysis was performed using Stata ver. 15 (Stata Corp).

## Results

In total, 105 patients were enrolled in the analysis. Of these, 40 (38.1%) were female. The mean age was 52 ± 14 years. Other baseline characteristics are shown in Table 1. We found that 64 patients (61%) had a defined source of infection, and 36 (34.3%) were intravenous drug users. The majority of the patients in the cohort, 74 (70.5%), had bacteremia due to methicillin-sensitive *S. aureus*, while 31 (29.5%) were methicillin-resistant *S. aureus*–related.

**Table 1:** Baseline characteristics of the study population (n = 105)

Characteristic	Value
Age (yr)	52 ± 14
Sex	
Female	40 (38.1)
Male	65 (61.9)
Diabetes	23 (21.9)
Hypertension	36 (34.3)
Dyslipidemia	15 (14.3)
Renal insufficiency	24 (22.9)
MSSA	74 (70.5)
MRSA	31 (29.5)
VIRSTA score	5.3 ± 2.1
More than moderate valvular regurgitation	11 (10.5)
Period of treatment (wk)	4.3 ± 2.5
Treated with valvular intervention	7 (6.7)
Mortality	8 (7.6)
Vegetation dimension (mm)	18 ± 9

Values are presented as mean ± standard deviation or number (%)

MSSA, methicillin-sensitive *Staphylococcus aureus*; MRSA, methicillin-resistant *Staphylococcus aureus*

Sixteen patients (15.2%) were diagnosed as confirmed IE: eight cases of tricuspid IE, four cases of mitral valve IE, three cases of aortic valve IE, and one patient with double valve involvement (mitral and tricuspid valves). The total in-hospital mortality was 7.6%, comprising two patients with IE and six patients without IE. In addition, one patient survived a cardiac arrest.

**Fig. 2:**
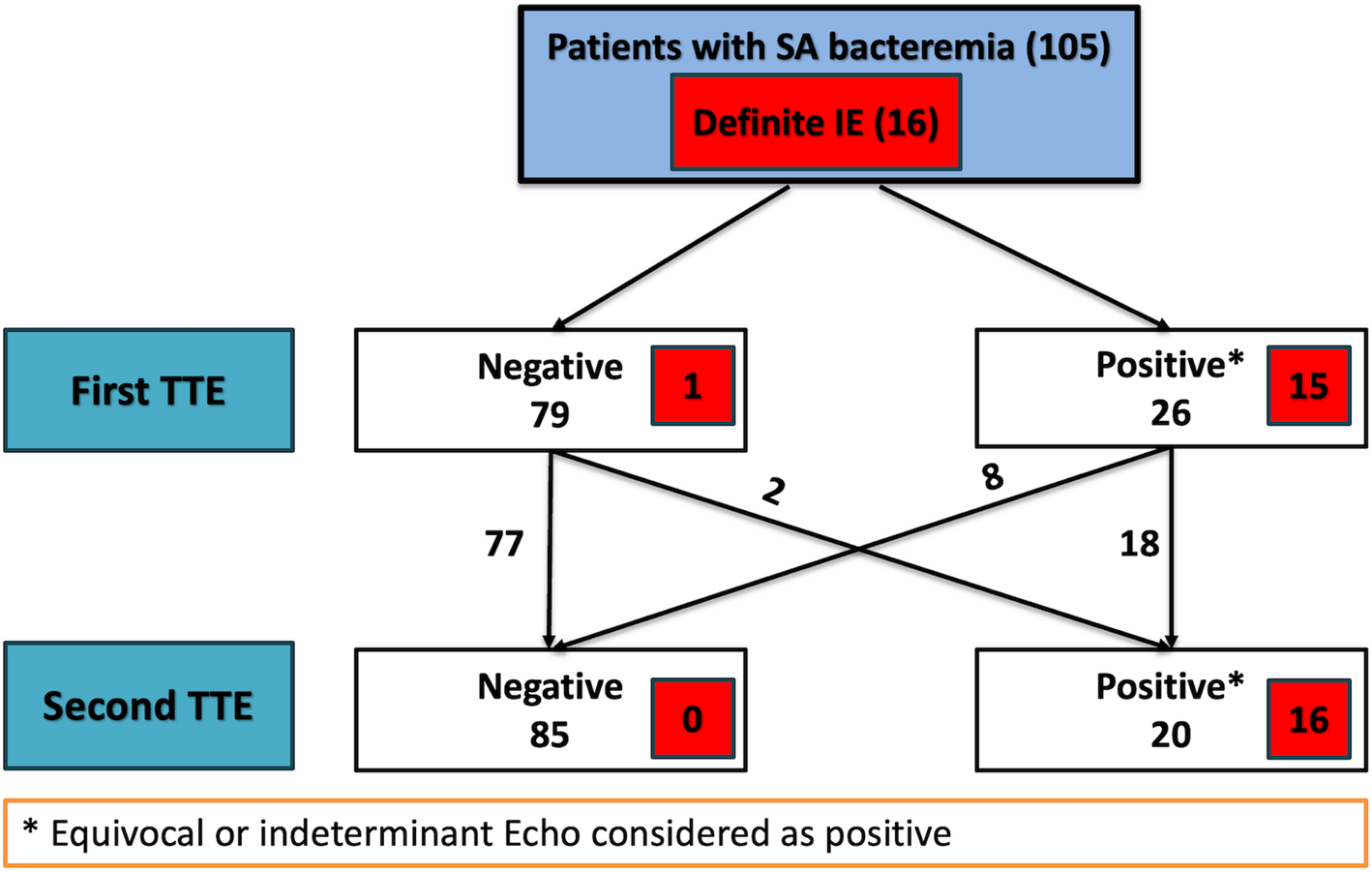
First and second transthoracic echocardiography (TTE) diagnostic results for detecting evidence of infective endocarditis (IE). Red boxes indicate the number of patients with definite IE. Among 105 patients with suspected IE, 16 were diagnosed with definite IE. Of these, one patient was misdiagnosed with the first TTE but correctly diagnosed with the second TTE. Patients with indeterminate or equivocal TTE results were classified as positive for IE. Poor echo window is defined as inadequate image quality, preventing clear visualization of all cardiac valves and structures. SAB, *Staphylococcus aureus* bacteremia

Only one patient with vegetation on TEE was missed on the first TTE but was detected with the second TTE. The first TTE identified echocardiographic evidence of IE with 93.8% sensitivity, 87.6% specificity, and 88.6% accuracy. Adding a second TTE yielded a sensitivity of 100%, a specificity of 95.5%, and a diagnostic accuracy of 96.2% in the detection of IE, as shown in Table 2 and Fig. 2.

**Table 2:** Diagnostic performance of the initial TTE and the additive findings of both TTEs

Statistics	First TTE	Second TTE
Sensitivity (%) (95% CI)	93.8 (69.8–99.8)	100 (79.4–100)
Specificity % (95% CI)	87.6 (78.9–93.7)	95.5 (88.9–98.8)
Positive predictive value (%) (95% CI)	57.7 (43.6–70.6)	80.0 (60.6–91.3)
Negative predictive value (%) (95% CI)	98.8 (92.1–99.8)	100 (95.9–100)
Accuracy (%) (95% CI)	88.6 (80.8–93.9)	96.2 (90.5–98.9)

TTE, transthoracic echocardiography; CI, confidence interval

Interobserver agreement was quantified using Cohen κ statistic. Excellent agreement was demonstrated (κ = 0.94). Notably, none of the 16 IE-positive cases were missed.

The duration of antibiotic treatment ranged from 1 to 8 weeks, with an average of 4.2 ± 2.1 weeks. In our cohort, 47 patients (44.7%) had a second positive blood culture obtained within 12 ± 3 days of the initial culture. During a 3-month follow-up period after hospital discharge, three patients were readmitted with recurrent bacteremia, one with acute renal failure, and one with hyperglycemia. However, none were readmitted or diagnosed with IE.

## Discussion

Our study is the first to prospectively investigate the additive value of repeating TTE within 1 week in patients with SAB. The findings demonstrate that performing a second TTE within this interval significantly improves diagnostic accuracy, sensitivity, and specificity for detecting IE in hospitalized patients with SAB. This suggests that repeated TTE offers a noninvasive and reliable alternative to TEE in this clinical setting.

TEE offers a superior diagnostic yield compared to TTE in detecting small and highly mobile structures, such as vegetations, abscesses, or fistulae, due to the shorter distance of the TEE probe to the cardiac structures and its higher temporal and spatial resolution [15]. Therefore, TEE is considered the gold standard tool for diagnosing IE. However, TEE is limited by resource availability and is a semi-invasive procedure with non-negligible risks related to the sedation used or the procedure itself [16, 17].

Due to its noninvasive nature and ready availability, TTE is the preferred diagnostic modality and should be performed as soon as possible to assess vegetations, valvular lesions, and complications in patients with suspected IE. A few studies have demonstrated that a normal TTE is useful for excluding IE in patients with SAB, with an NPV of approximately 95% [8, 18]. Additionally, Van Hal et al. [5] found that the NPV of TTE can exceed 98% when there is normal or trivial valvular regurgitation, especially in patients without embolic phenomena, particularly when TTE is performed 5 or more days after the initial SAB diagnosis. These findings align with the NPV observed with the initial TTE in our study (98.8%). Moreover, including a repeat TTE improves the NPV to 100%.

Multiple studies compared the diagnostic utility of TTE to TEE in identifying *S. aureus*–related IE. In a prospective study of 103 patients with SAB, Fowler et al. [4] found that TTE had a sensitivity of only 32% and a specificity of 100% for detecting IE. Similarly, Sekar et al. [19] reported a significantly lower sensitivity of TTE (24%) in comparison to TEE (94%), with a P-value of < 0.001. These findings contrast with our results, where the initial TTE demonstrated a markedly higher sensitivity (93.8%) and specificity (87.6%). This difference may be attributed to patient selection (excluding patients with intracardiac devices and patients with poor echo windows, which are associated with inadequate image quality, preventing clear visualization of all cardiac valves and structures), timing of the initial TTE, operator experience, or advances in echocardiographic technology.

**Fig. 3:**
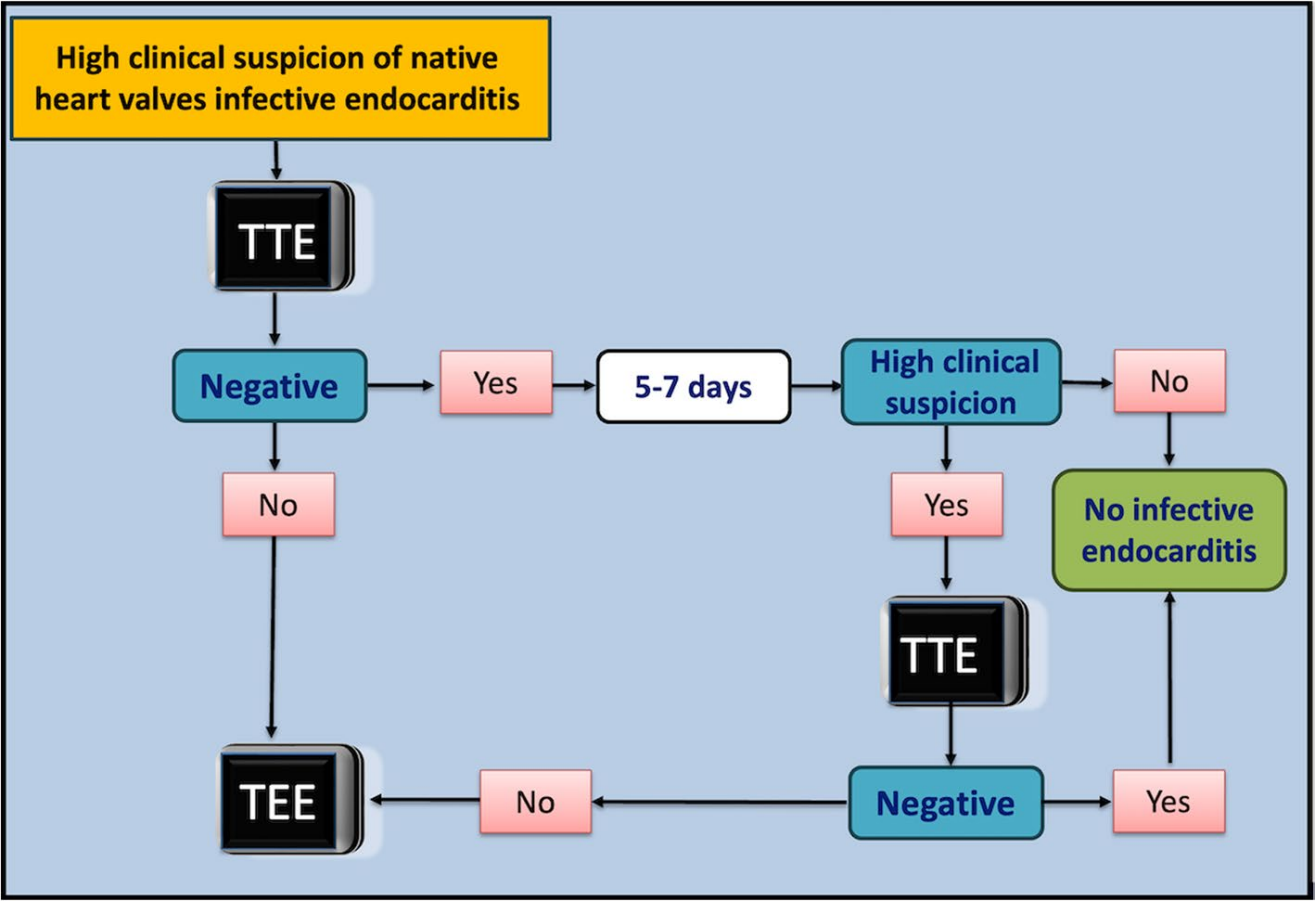
Diagnostic approach for patients with high clinical suspicion of native valve infective endocarditis. TEE, transoesophageal echocardiography; TTE, transthoracic echocardiography

In contrast, Lindner et al. [9] demonstrated an 83% agreement between TEE and TTE in determining the likelihood of IE among patients with interpretable echocardiographic studies. These findings are consistent with our results, which also demonstrated a high level of concordance between TTE and TEE when both were performed within a 1-week interval. Furthermore, the addition of a second TTE markedly improved the sensitivity to 100%, specificity to 95.5%, and, importantly, NPV to 100%. This significant enhancement underscores the importance of repeated imaging to capture evolving pathologic changes, which might not be evident on initial examination. Based on our study results, we propose an algorithm for the diagnostic approach to patient SAB and suspected IE, as outlined in Fig. 3.

In this study, we observed a high diagnostic accuracy of echocardiography, probably due to the selective inclusion of patients with native valve endocarditis and the exclusion of those with cardiac devices. Similar findings were reported by Gomes et al. [20], who found echocardiographic specificity of 100%, a positive predictive value of 100%, and an NPV of 91% among patients with native valve endocarditis.

Echocardiography is considered the first-line imaging modality for IE. However, multimodality imaging, including nuclear imaging and computed tomography, is increasingly being used. Especially in the case of patients with prosthetic material or indwelling catheters, due to the limitations of echocardiography in these circumstances [20–22].

Our study has multiple limitations. First, the sample size is relatively small. Second, given the low incidence of IE overall (16 of 105, 15.2%), enrolling a large number of confirmed cases within a single-center prospective study is inherently challenging. Third, the study was conducted at a single center. Fourth, patients with a history of IE, valve interventions, cardiac devices, or intracardiac or dialysis catheters were excluded due to the limited diagnostic value of TTE in these cases. As a result, our findings may not be applicable to this group of patients. Finally, although repeated TTE demonstrated high diagnostic accuracy in this study, we did not assess whether it influenced clinical decision-making, as all patients underwent TEE regardless of TTE findings. Future studies comparing TTE-based and TEE-based strategies are necessary to determine the real-world clinical utility of repeated TTE.

## Conclusions

Repeating TTE within 5 to 7 days demonstrated significant diagnostic value in identifying IE in patients with SAB and native valves. This strategy yielded a high NPV for excluding IE, suggesting that this strategy may reduce, but not replace, the need for TEE in selected low-risk patients. However, given that TEE remains the gold standard, particularly for detecting complications such as abscesses or in high-risk subgroups (e.g., prosthetic valves, intracardiac devices), caution is warranted in generalizing these findings. Larger, multicenter studies are needed to validate this approach and define its role within updated clinical pathways.

## Data Availability

No datasets were generated or analysed during the current study.

## Abbreviations

IE: Infective endocarditis
NPV: Negative predictive value
SAB: *Staphylococcus aureus* Bacteremia
TEE: Transoesophageal echocardiography
TTE: Transthoracic echocardiography
